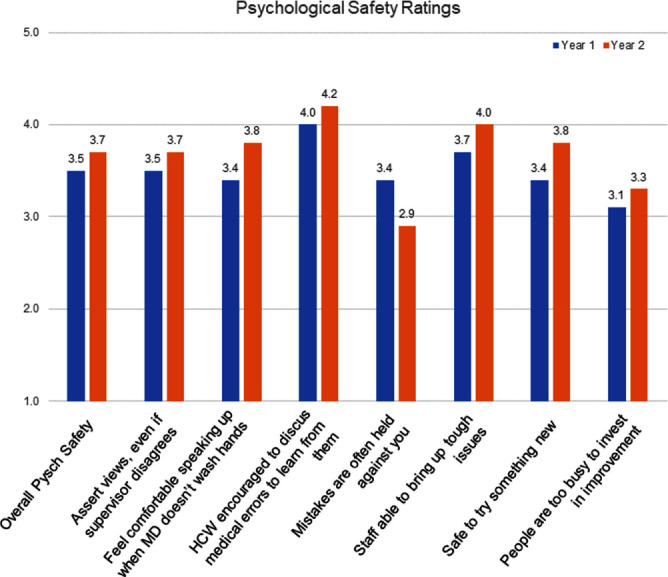# Low levels of psychological safety in inpatient medical-surgical nurses on the tail end the COVID-19 pandemic

**DOI:** 10.1017/ash.2024.292

**Published:** 2024-09-16

**Authors:** Cara Johnson, Ulanda Marcus-Aiyeku, Amanda Hessels

**Affiliations:** Columbia University School of Nursing; Hackensack Meridian Health, University of Tennessee Health Science Center; Columbia University

## Abstract

**Background:** The COVID-19 pandemic required an increased reliance on and utilization of the inpatient nursing workforce. We aim to examine the psychological safety of U.S. inpatient acute care nurses in the two years following the onset of COVID-19. **Method:** Participants were recruited for participation across two major metropolitan medical centers in the tristate area, six units per site (12 total). Anonymous Qualtrics survey invitations were distributed through work listservs in the first halves of 2022 and 2023. The invitation was open to registered nurses who provided at least 16 hours/week of direct patient care for at least six months at the hospital. The survey was open for 4-6 weeks, with reminders sent every other week. Nurses were offered a $25 gift card the firs year and $35 the second. Nurses were asked to rate how frequently they experience seven conditions indicative of psychological safety in their work environment from 1 = “Never” to 5 = “Always”. Two items were reverse coded in analysis as the prompts were negatively phrased. Blank and ineligible responses were excluded from the analytic sample. As response distributions were skewed, Wilcoxon rank-sum tests were used to analyze differences between years (alpha = 0.5). Blank and ineligible responses were excluded from the final analytic sample. **Result:** We achieved an overall response rate of 52% for each survey year (n=258 Year 1, n=221 Year 2). Psychological safety was found to be low overall for both years, but lowest for Year 1 (3.5 vs. 3.7, p=0.0132). The highest rated condition in both years was “When a medical error occurs at this hospital, health care workers are encouraged to discuss mistakes in order to learn how to prevent similar future errors” (4.0 Year 1 vs. 4.2 Year 2, p=0.0054). The lowest rated condition changed across years. For Year 1, “At this hospital, people are too busy to invest time in improvement” (reverse coded) received the lowest rating at 3.1 (vs. 3.3 in Year 2, p=0.0685). For Year 2, “If you make a mistake at this hospital, it is often held against you” became the lowest rating at 2.9 (vs. 3.4 year 1, <.0001). A summary of the psychological safety conditions is presented in Figure 1. **Conclusion:** Nurses in our study reported low psychological safety during the end of the COVID-19 pandemic. This has implications for overall patient safety and for nursing staff retention in acute care units.

**Figure:**